# Impact of antimicrobial stewardship on antibiogram, consumption and incidence of multi drug resistance

**DOI:** 10.1186/s12879-022-07906-1

**Published:** 2022-12-07

**Authors:** Rula M. Darwish, Sajeda Ghassan Matar, Ahmad Atef Abu Snaineh, Mohammad Refat Alsharif, Ahmad Bassam Yahia, Haneen Nidal Mustafa, Elfatih A. Hasabo

**Affiliations:** 1 Department of Pharmaceutics and Pharmaceutical Technology, School of Pharmacy, The University of Jordan, Amman, Jordan; 2 Faculty of Pharmacy, Applied Science Private University, Amman, Jordan; 3 Istishari Hospital, Amman, Jordan; 4 Istiklal Hospital Lab, Istiklal Hospital, Amman, Jordan; 5 Jordanian Ministry of Health, Amman, Jordan; 6 Faculty of Medicine, University of Khartoum, Khartoum, Sudan; 7 School of Pharmacy, The University of Jordan, Amman, Jordan

**Keywords:** Antimicrobial stewardship program, Antibiotics, Antibiotic resistance, Multi-drug resistance, ESBL *E. coli*, ESBL *Klebsiella*, MRSA, VRE, Defined Daily Dose, Antibiogram

## Abstract

**Introduction:**

Antimicrobial stewardship programs are intended to improve patient outcomes, reduce side effects, bacterial resistance, and costs. Thus, it is important to assess their impact on an ongoing basis. We aimed to assess the impact of the antimicrobial stewardship program in two different hospitals which used different program approaches.

**Methodology:**

This is a retrospective observational study in two private hospitals [4088 patient records] in Amman- Jordan. Antibiotic susceptibility using antibiogram results, consumption of antibiotics using Defined Daily Dose, and the incidence of Multi-Drug Resistance were recorded using patients’ records during 2018, 2019, and 2020.

**Results:**

Antimicrobial stewardship program outcomes varied between the two hospitals. Bacterial susceptibility to antibiotics were improved in both hospitals. Moreover, the defined daily dose in Hospital “A” showed no significant change in Fluoroquinolones, Carbapenems, and Piperacillin- Tazobactam, Cephalosporins, and Colistin, while a significant change was observed among Anti-MRSA antibiotics. Finally, the incidence of Extended Spectrum Beta-lactamase [ESBL] *E. coli*, ESBL *Klebsiella*, and Vancomycin Resistant Enterococci [VRE] have decreased numerically over the study period, while Methicillin-Resistant *Staphylococcus aureus* [MRSA] showed an increase in incidence during the second year of the study.

**Conclusion:**

The study emphasizes the positive impact of the AMS program throughout the three years of the study. Plus, the need to enhance the program through recruiting extra staff and applying extra regulations like implementing educational programs for the hospital staff, designing local guidelines for common ID diseases, and monitoring the program’s outcomes which would eventually be more efficient, cost-effective, and safe.

## Strengths

- This study is one of the first studies which assess antimicrobial stewardship program in the region, and the first in Jordan which is considered to be the first step toward protecting antibiotics from extinction by finding new measurements to decrease resistance.
- This study has assessed antimicrobial stewardship programs objectively in two hospitals and concluded some realistic solutions and measurements to practice enhancing the program outcomes.

## Limitations

- Only two hospitals were included in this study. The more the hospitals participate, the more antimicrobial stewardship experience is densified and the more we learn.
- Defined Daily Dose (DDD) was only calculated in Hospital “A”. On the other hand, we could not find data to calculate in Hospital “B”.
- This study lasted for 3 years (2018–2020), which means that one year of the COVID-19 pandemic was included, accompanied by changes in infection control policies that may possibly alter the incidence of multidrug resistance.

## Introduction

Antibiotics have extended life spans worldwide, and have significantly contributed to morbidity and mortality reduction from dangerous infections [1]. However, the overuse of antibiotics drives the evolution of resistance which would result to “hard-to-treat”, and often untreatable infections, which are now considered a worldwide challenge [2]. Antibiotic resistance leads to ineffective therapy, forcing health care providers to use more toxic agents for longer durations which increases the overall cost [3]. For instance, the overuse of beta-lactam antibiotics have expanded the occurrence of carbapenem-resistant pathogens [4]. The Centers for Disease Control and Prevention [CDC] estimated that around 30% of antibiotics used in hospitals are inappropriate or unnecessary [5].

In Jordan, the prevalence of self-medication with antibiotics is significantly high [6]. Another study showed that more than 50% of physicians prescribe antibiotics for inappropriate indications such as, the common cold. Approximately, 67% of adult Jordanians believe that antibiotics should be used for this purpose. 28.1% misused antibiotics as analgesics. 49.0% use left-over antibiotics without physicians’ consultation [7].

As antibiotic resistance continues to rise, healthcare facilities should adopt preventive measures to contain the problem. This would include antimicrobial stewardship programs [AMSs], and infection control programs [ICPs]. These programs are essential since they would help monitor antimicrobial use, That would lead to improvement in patient outcomes, control antibiotics prescription, optimize the quality of care among infected patients, and reduce healthcare as well as, societal costs [8].

In this study, we aim to assess the impact of AMS implementation on antibiotic susceptibility, as well as on DDD in two different hospitals [which are using different approaches for the program] in Amman- Jordan.

## Materials and methods

### Study design

The study is a retrospective study, designed according to STROBE guidelines for observational studies [9], that recorded all culture results one year before the initiation of antimicrobial stewardship [2018] and 2 years after the initiation of the antimicrobial stewardship program [2019 and 2020]. Two hospitals participated in this study; they will be referred to as hospitals “A” and “B”. Hospital “A” is a private hospital in Amman- Jordan consisting of 110 beds, a gynecology department, surgery, internal medicine department, bone marrow transplant unit, intensive care unit, neonates intensive care unit, and in vitro fertilization department. Hospital “B” is a private hospital as well; it has 280 beds, in vitro fertilization department, ear nose, and throat department, hematology, and oncology unit, lithotripsy unit, kidney transplant unit, cosmetology and hair transplantation unit, cardiovascular surgery unit, internal and surgery department, intensive care unit, and neonates’ intensive care unit. A total of 4088 patients were included in this study; 2405 patients from Hospital “A” [1038 during 2018, 686 during 2019, and 681 during 2020] and 1683 patients from Hospital “B” [579 during 2018, 593 during 2019, and 511 during 2020].

### Structure of antibiotic stewardship team

In Hospital “A”, the team of the stewardship program included an Infectious disease consultant [ID], a clinical pharmacist, a microbiology lab technician, an infection control nurse, and a quality control department staff member. On the other hand, Hospital “B” team comprises an ID specialist, medical director, pharmacy director, clinical pharmacist, lab director, microbiology technician, nursing director, infection control nurse, quality director, and information technology [IT] director.

### Antimicrobial stewardship implementation

Strategies to decrease antibiotic resistance varied between the two hospitals. Hospital “A” listed restricted antibiotics into three categories, the first one includes those which are only allowed to be prescribed by an ID consultant. The second contains those which are prescribed by an ID consultant plus a transplant consultant, and the third comprises those restricted for ID consultant plus a pulmonologist plus an oncologist (Table 1). In addition to that, Hospital “A” created its local guidelines for antibiotics selection, doses, and duration for most common bacterial infectious diseases, including community-acquired pneumonia, hospital-acquired pneumonia [HAP], and urinary tract infection [UTI]. While Hospital “B”, followed a restrictive strategy in which some identified antibiotics are only allowed to be prescribed after the approval of the ID consultant (Table 1), moreover, Hospital “B” created a policy to automatically stop all antibiotics’ prescribed on day seven unless a consultant ordered otherwise. Both hospitals monitored antibiogram results annually, while only Hospital “A” monitored Defined Daily Dose [DDD].

**Table 1:** List of restricted antimicrobial agents for all three participating hospitals

		Hospital “A”	Hospital “B”
Restricted antibiotics	Antibiotics restricted to infectious disease consultant	- Colistin - Anti-materials drugs - Anti-tuberculosis drugs (except for pulmonologists for TB patients) Any new antimicrobial agents	- Linezolid - Ceftolozane/tazobactam - Ceftoboprol/ medocaril - Ceftaroline - Colistimethate
	Antimicrobials restricted to infectious diseases and transplant consultant:	- Ganciclovir - Valganciclovir - Daptomycin - Cidovir - Foscarnet - Pentamidine I. V	–
	Antimicrobial restricted to infectious disease PLUS pulmonologist PLUS oncologist	- Linezolid - Abelcet - Voriconazole - Posaconazole - Echinocandins	–

### Outcomes measured

Three main outcomes were used to assess the efficacy of the antimicrobial stewardship program; the change in antibiotic susceptibility according to antibiograms, the consumption of antibiotics according to the Defined Daily Dose [DDD] per one thousand patients, and the incidence of more resistant bacteria including ESBL *E. coli,* ESBL *Klebsiella,* MRSA, and VRE. Multidrug-resistant (MDR) is defined as the resistance toward at least one antibiotic in three different antibiotics classes or more, ESBL, MRSA, and VRE microorganisms are considered multidrug-resistant organisms [10, 11].

### Laboratory methods

Hospital “A” and Hospital “B” used the zone of inhibition test to determine susceptibility according to the Clinical & Laboratory Standards Institute [12]. All specimens received with a full name, age, and gender, urine samples are cultured on blood and MacConkey media, then incubated at 37 Celsius for 18–24 h while swabs from different sources [wound, puss, cerebrospinal fluid, semen] are cultured on blood, MacConkey, chocolate agar and sabouraud dextrose agar for 18–24 h, when finding a bacterial growth, identification and diagnosis of bacteria are performed. Identification protocol includes citrate agar test, bile esculin test, SIM agar test, urea test, triple sugar iron test, and API system susceptibility test on Mueller Hinton agar using the selective types of antibiotics according to the type of bacterial growth All agar media including MacConkey, Blood agar, Citrate agar, SIM agar, and Muller Hinton agar were all used from OXOID company for microbiological laboratory supplements—UK. Bacterial culture results were collected by the infection control department in each hospital and were analyzed to create an antibiogram annually.

### Defined Daily Dose [DDD]

Defined Daily Dose [DDD] is a measure of antibiotics consumption. It is promoted by the World Health Organization [WHO], and is widely used in antimicrobial stewardship programs [13]. It was measured per one thousand patients using WHO guidelines for each antibiotic.

### Statistics

Statistical analysis were performed using SPSS version 26. McNemar’s test was used to calculate the P-value for the change in antibiotic susceptibility before and after antimicrobial stewardship, and the Kruskal-Wallis test was used to calculate the P-value for the change in DDD per 1000 patient per class of antibiotics, a P-value < 0.05 was considered significant.

### Ethical approval

Ethical approval from the hospitals was obtained as an institutional policy for the research, and approval for participating in this study from both hospitals in [August 2020].

## Results

### Change in antibiotic susceptibility

The percentage of susceptibility of antibiotic towards different bacteria which have changed through AMS in Hospital “A” and “B”, are shown in Tables 2 and 3; respectively. When comparing results between one year before the implementation of the AMS program and one year after, six cases have significantly improved percentages of susceptibility in Hospital “A” while two cases showed a significant decrease in susceptibility in the same hospital, while seven cases showed a significant increase in susceptibility with sixteen cases demonstrating decreased susceptibility in Hospital “B” when comparing results between one year before implementation of AMS program and one year after. After the first year, while comparing results of the first year and the second year of implementing the AMS program, three cases showed significantly improved percentages of susceptibility in Hospital “A” and only one case of decreased antibiotic susceptibility. On the other hand, Hospital “B” showed twenty cases of improved antibiotic susceptibility and seven cases of decreased susceptibility, Table 3 summarizes antibiotics that have changed significantly in percentages of susceptibility. Many antibiotics showed no change [numerical or significant] over the years of this study; Hospital “A” showed 83 cases of constant results of the percentages of antibiotic susceptibility, while Hospital “B” showed 126 constant results of the percentages of antibiotic susceptibility. Table 4 shows a summary of antibiotics that have a change in percentages of susceptibility throughout this study.

**Table 2:** Percentage of antibiotics’ sensitivity toward different bacteria in Hospital “A” before and after the implementation of Antimicrobial Stewardship Program

Bacteria	Number of cultures			Percentage of sensitive cultures for each antibiotic				P-value to compare between pre and one year after	P-value to compare between one year and two years after
	One year before application of AMS program	One year after application of AMS program	Two year after application of AMS program	One year before application of AMS program	One year after application of AMS program	Two year after application of AMS program			
*E. coli*	456	355	324	Piperacillin/tazobactam				0.632	0.461
				80	82	85			
				Cefuroxime				–	0.477
				48	48	50			
				Ceftazidime				0.7	0.705
				48	49	50			
				Ertapenem				** < 0.001**	** < 0.001**
				93	69	94			
				Imipenem				** < 0.001**	** < 0.001**
				93	69	94			
				Gentamycin				**0.002**	0.709
				69	79	77			
				Amikacin				0.077	0.18
				97	99	97			
				Ciprofloxacin				1	1
				53	54	53			
				Tigecycline				0.405	–
				95	97	97			
				TMP/SMX				1	0.74
				41	43	45			
*Klebsiella pneumonia*	140	75	88	Piperacillin/tazobactam				0.442	0.542
				63	68	76			
				Cefuroxime				0.627	–
				45	51	51			
				Ceftazidime				0.755	0.864
				42	48	51			
				Ertapenem				**< 0.001**	**< 0.001**
				79	84	86			
				Imipenem				1	0.815
				79	84	86			
				Gentamycin				0.229	0.152
				75	64	76			
				Amikacin				0.581	0.774
				92	89	91			
				Ciprofloxacin				**0.006**	0.458
				47	73	69			
				Tigecycline				0.405	–
				81	84	90			
				TMP/SMX				0.533	0.652
				56	57	51			
*Pseudomonas aeruginosa*	95	41	51	Piperacillin/tazobactam				0.629	0.07
				75	80	94			
				Ceftazidime				–	0.774
				78	78	84			
				Imipenem				1	0.092
				62	71	82			
				Gentamycin				–	0.424
				73	73	86			
				Amikacin				0.774	0.754
				84	83	88			
				Ciprofloxacin				0.096	0.503
				67	56	69			
*Acinetobacter*	65	20	26	Tigecycline				0.227	0.388
				69	50	65			
*Enterobacter*	33	20	12	Piperacillin/tazobactam				0.549	
				54	60	100			
				Cefuroxime				0.18	0.18
				36	50	83			
				Ceftazidime				0.289	0.219
				36	50	83			
				Ertapenem				1	0.375
				72	75	99			
				Imipenem				1	0.625
				72	75	99			
				Gentamycin				0.289	0.625
				63	80	99			
				Ciprofloxacin				0.344	1
				54	80	75			
				TMP/SMX ( Trimethoprim / Sulfamethoxazole)				**0.004**	0.375
				27	65	50			
*Proetus mirabilis*	30	21	15	Cefuroxime				0.727	1
				73	76	67			
				Piperacillin/tazobactam				0.549	1
				54	60	100			
				Ceftazidime				1	1
				73	76	67			
				Imipenem				1	1
				6	10	13			
				Gentamycin				0.727	0.462
				53	57	60			
				Amikacin				0.625	1
				93	86	93			
				Ciprofloxacin				0.581	1
				50	62	76			
				TMP-SMX				1	1
				36	43	47			
*S. aureus*	97	81	80	Oxacillin				0.337	0.87
				39	31	29			
				Piperacillin/tazobactam					0.86
					31	29			
				Gentamycin				0.458	0.071
				87	88	85			
				Ciprofloxacin				0.093	**0.004**
				72	79	64			
				TMP-SMX ( Trimethoprim / Sulfamethoxazole)				0.629	0.815
				88	77	94			
				Clindamycin				0.487	1
				61	65	66			
				Rifampin				0.388	0.549
				91	95	91			
*E. faecalis*	48	30	30	Ciprofloxacin				0.087	–
				54	60	60			
				Vancomycin				**0.002**	0.871
				87	93	100			
				Teicoplanin				**< 0.001**	0.851
				87	93	100			
*E. faecium*	22	2	10	Ciprofloxacin				1	1
				18	50	36			
*Strep. Viridians*	25	15	11	Ciprofloxacin				0.727	1
				60	70	80			
				Piperacillin/tazobactam				–	1
					75	80			
Total number of cases with improved antibiotics’ sensitivity				When comparing 1 year before AMS and 1 year after: **6**			When comparing 1 year after and 2 years after AMS: **3**		
Total number of cases with decreased antibiotics’ sensitivity				When comparing 1 year before AMS and 1 year after: **2**			When comparing 1 year after and 2 years after AMS: **1**		

Statistically significant values are shown in bold

**Table 3:** Percentage of antibiotics’ sensitivity toward different bacteria in Hospital “B” before and after the implementation of Antimicrobial Stewardship Program

Bacteria	Number of cultures			Percentage of sensitive cultures for each antibiotic			P-value to compare between pre and one year after	P-value to compare between one year and two years after
	One year before application of AMS program	One year after application of AMS program	Two year after application of AMS program	One year before application of AMS program	One year after application of AMS program	Two year after application of AMS program		
ESBL *E. coli*	124	156	126	TMP/SMX ( Trimethoprim / Sulfamethoxazole)			–	1
				22	22	26		
				Ciprofloxacin			0.88	–
				23	24	24		
				Piperacillin-tazobactam			0.291	0.291
				79	73	79		
*E. coli*	126	177	149	Ampicillin			0.549	0.549
				3	5	3		
				Amoxicillin—clavulanate			0.583	0.32
				57	53	59		
				Cefuroxime			**0.02**	**0.027**
				70	59	70		
				Ciprofloxacin			0.609	0.068
				55	48	59		
				TMP/SMX			0.807	0.336
				52	49	54		
				Piperacillin-tazobactam			**0.012**	0.185
				100	93	87		
				Cefalexin			**< 0.001**	0.487
				48	14	17		
				Levofloxacin				0.081
					48	59		
ESBL *Klebsiella*	59	32	32	Ciprofloxacin			0.21	1
				20	47	50		
				TMP/SMX ( Trimethoprim / Sulfamethoxazole)			1	1
				10	9	13		
				Piperacillin-tazobactam			1	1
				69	66	69		
				Levofloxacin			–	1
					47	50		
*Klebsiella* spp	90	77	81	Amoxicillin -clavulanate			**0.019**	0.678
				7	21	27		
				Amikacin			**< 0.001**	**0.014**
				83	55	75		
				Gentamycin			**< 0.001**	1
				70	0	1		
				Cefuroxime			**< 0.001**	0.868
				70	34	37		
				Ceftazidime			**0.001**	**0.009**
				70	39	59		
				Ceftriaxone			0.08	**0.04**
				23	39	59		
				Ciprofloxacin			0.136	0.728
				22	32	35		
				TMP/SMX			**< 0.001**	0.864
				76	22	25		
				Ertapenem			**0.035**	**0.017**
				70	53	74		
				Piperacillin-tazobactam			**0.047**	**0.017**
				70	53	74		
				Imipenem			0.09	**0.032**
				70	53	74		
				Meropenem			0.065	**0.014**
				70	53	74		
				Tigecycline			0.099	**0.032**
				30	47	26		
				Cefoxitin			** < 0.001**	–
				24	1	1		
				Cefalexin			** < 0.001**	–
				70	6	6		
				Colistin			0.081	**0.014**
				30	47	26		
				Levofloxacin			0.125	** < 0.001**
				0	8	35		
*Pseudomonas aeruginosa*	65	41	26	Gentamycin			**0.002**	1
				89	63	69		
				Ceftazidime			0.453	0.219
				85	90	73		
				Ciprofloxacin			** < 0.001**	1
				54	95	92		
				TMP/SMX			1	–
				3	1	1		
				Piperacillin-tazobactam			** < 0.001**	1
				52	95	96		
				Imipenem			**0.017**	1
				57	88	92		
				Meropenem			**0.001**	0.687
				57	78	92		
				Tigecycline			** < 0.001**	**0.011**
				5	49	77		
				Levofloxacin			–	1
					94	92		
				Colistin			1	1
				94	93	92		
*Acinobacter baumanii*	51	56	50	Amikacin			0.625	1
				2	9	6		
MRSA	7	3	19	Gentamycin			1	1
				14	67	89		
				Ciprofloxacin			1	1
				14	33	84		
				TMP/SMX ( Trimethoprim / Sulfamethoxazole)			1	1
				57	33	79		
				Piperacillin-tazobactam			1	1
				43	33	95		
				Levofloxacin			–	1
					33	84		
				Erythromycin			1	1
				14	33	26		
				Clindamycin			1	1
				14	33	26		
*Strep. faecalis*		15	13	Ampicillin			–	1
					26	31		
				Amoxicillin-clavulanate			–	0.508
					47	62		
				Amikacin			–	**0.021**
					1	69		
				Gentamycin			–	**0.07**
					1	54		
				Cefuroxime			–	**0.021**
					1	77		
				Ceftazidime			–	**0.003**
					1	92		
				Ceftriaxone			–	**0.003**
					1	92		
				Ciprofloxacin			–	0.687
					53	77		
				TMP/SMX			–	**0.039**
					33	85		
				Ertapenem			–	**0.008**
					20	85		
				Piperacillin-tazobactam			–	0.727
					47	77		
				Imipenem			–	**0.008**
					27	85		
				Meropenem			–	**0.002**
					20	92		
				Tigecycline			–	** < 0.001**
					7	100		
				Levofloxacin			–	0.125
					53	85		
*Staphylococcus aureus*	55	36	15	Ampicillin			0.625	0.625
				0	8	20		
				Amoxicillin-Clavulanate			**0.006**	0.375
				100	69	53		
				Amikacin			**0.006**	**0.004**
				100	69	0		
				Gentamycin			0.481	**0.021**
				75	61	0		
				Cefuroxime			** < 0.001**	** < 0.001**
				20	86	0		
				Ceftazidime			1	0.07
				35	36	0		
				Ceftriaxone			1	**0.016**
				25	36	0		
				Ciprofloxacin			0.581	0.289
				44	61	47		
				TMP/SMX			0.21	0.18
				40	64	33		
				Ertapenem			**< 0.001**	1
				100	61	27		
				Piperacillin-Tazobactam			1	0.453
				53	58	47		
				Tigecycline			–	**< 0.001**
					100	13		
				Cefalexin			0.286	0.125
				33	44	0		
				Levofloxacin			–	0.289
					61	47		
				Erythromycin			0.824	0.219
				51	47	1		
				Clindamycin			0.607	0.289
				51	47	13		

Statistically significant values are shown in bold

**Table 4:** Summary of antibiotics that have change in percentages of sensitivity

Hospital “A”											
*When comparing one year before implementing AMS and one year after*						*When comparing the first and second year of implementing AMS*					
*Antibiotics that showed ****increase**** in antibiotics sensitivity*			*Antibiotics that showed ****decrease**** in antibiotics sensitivity*			*Antibiotics that showed ****increase**** in antibiotics sensitivity*			*Antibiotics that showed ****decrease**** in antibiotics sensitivity*		
*E. coli*	Gentamycin		*Klebsiella*	Ertapenem		*E. coli*	Ertapenem		*Staphylococcus aureus*		Ciprofloxacin
*Klebsiella*	Ciprofloxacin		*E. coli*	Ertapenem Imipenem			Imipenem				
Enterobacter	TMP/SMX										
*E. faecalis*	Vancomycin Teicoplanin					*Klebsiella*	Ertapenem				

Hospital “B”											
*When comparing one year before implementing AMS and one year after*						*When comparing the first and second year of implementing AMS*					
*Antibiotics that showed ****increase**** in antibiotics sensitivity*			*Antibiotics that showed ****decrease**** in antibiotics sensitivity*			*Antibiotics that showed ****increase**** in antibiotics sensitivity*			*Antibiotics that showed ****decrease**** in antibiotics sensitivity*		
*Staphylococcus aureus*		Cefuroxime	*Pseudomonas*		Gentamycin	*Pseudomonas aeruginosa*		Tigecycline	*Klebsiella*	Tigecycline Colistin	
			*E. coli*		Ciprofloxacin Piperacillin- tazobactam Cefalexin	*E. coli*		Cefuroxime			
*Klebsiella*		Amoxicillin- clavulanate				*Klebsiella*		Amikacin Ceftazidime Ceftriaxone Ertapenem Piperacillin- tazobactam Imipenem Meropenem Levofloxacin	*Staphylococcus aureus*	Amikacin Gentamycin Cefuroxime Ceftazidime Tigecycline	
*Pseudomonas aeruginosa*		Ciprofloxacin Piperacillin- tazobactam Imipenem Meropenem Tigecycline	*Staphylococcus aureus*		Amoxicillin- clavulanate Amikacin Ertapenem						
			*Klebsiella*		Amikacin Gentamycin Cefuroxime Ceftazidime TMP/SMX Ertapenem Piperacillin- tazobactam Cefoxitin Cefalexin	*Streptococcus aureus*		Amikacin Gentamycin Cefuroxime Ceftazidime Ceftriaxone TMP/SMX Ertapenem Imipenem Meropenem Tigecycline			

### Defined Daily Dose [DDD] per 1000 patients

Hospital “A” has calculated antibiotics’ consumption and DDD, Table 5 shows results of DDD/1000 patients for Hospital “A”, no significant change in Fluoroquinolones, Carbapenems, and Piperacillin- Tazobactam, Cephalosporins, and Colistin, while a significant change was observed among Anti-MRSA antibiotics since results shows that the consumption of anti-MRSA antibiotics has decreased significantly. DDD/1000 patient was not calculated in Hospital B due to lack of documentation.

**Table 5:** Defined Daily Dose (DDD/1000 patient) in Hospital A before and after initiation of AMS

Class of antibiotics	DDD/1000 patient		P-value
	2019	2020	
FQ	33.7	30.6	0.556
Carbapenems	66.8	68.8	0.706
Piperacillin	82	62.5	0.814
Colistin	20.8	21.8	0.814
Cephalosporins	35.5	32.8	0.58
Anti-MRSA	58	50.7	0.045

*MRSA* methicillin-resistant *Staphylococcus aureus*, *FQ* fluoroquinolones, *DDD* Defined Daily Dose

### Development of ESBL, VRE, and MRSA infections.

Percentage of development of ESBL *E. coli* and ESBL *Klebsiella* have decreased numerically through the three years of this study in both Hospital “A” and Hospital “B”. Average percentages of ESBL *E. coli* in Hospital “B” and “A” during the three years of this study was 47.4%, and 50.9%; respectively., while the average percentage of ESBL *Klebsiella* in Hospital “A” and “B” was 52.8% and 23.4%respectively.

When reporting Methicillin-Resistant *Staphylococcus aureus* [MRSA] percentages, Hospital “B” showed a slight numerical decrease in MRSA reports during the first year, while showing a major numerical increase in its percentage during the second year of implementing the AMS program. The average percentage of MRSA was 24.9% over the three years of the study.

**Fig. 1:**
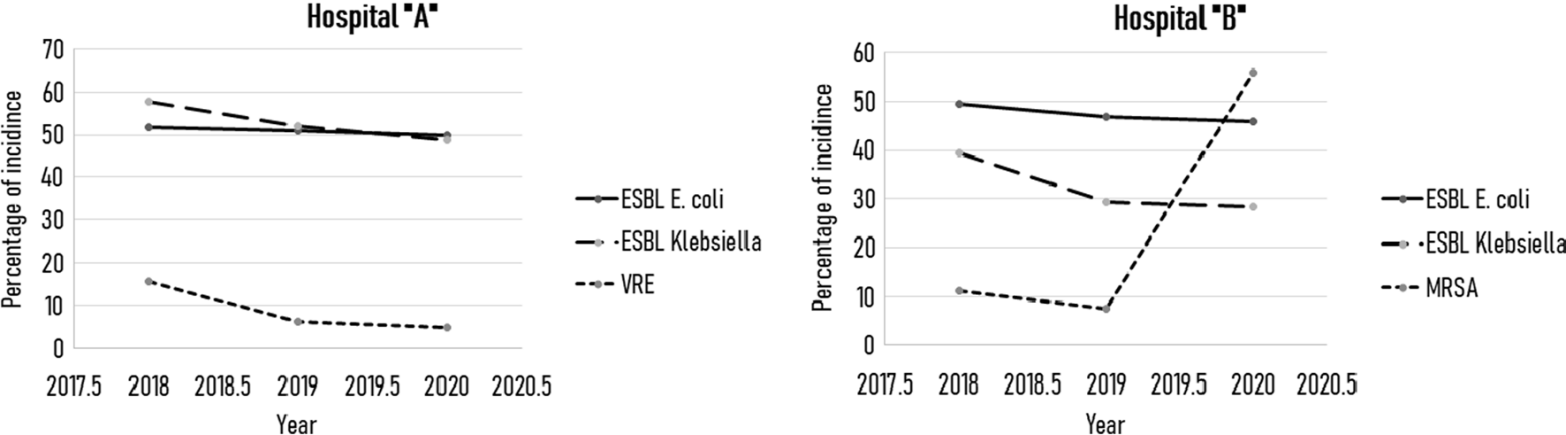
The change in ESBL *E. coli*, ESBL *Klebsiella,* VRE, and MRSA reports through the three years of the study

Vancomycin-Resistant Enterococcus [VRE] results in Hospital “A” showed a numerical decrease in its percentage over the three years of the study, the average percentage of VRE was 8.9%. Figure 1 shows the change in ESBL *E. coli*, ESBL *Klebsiella,* VRE, and MRSA reports through the 3 years of the study.

## Discussion

The first prospective audit for the antibiotic-streamlining program was initiated in 1988 at Hartford Hospital, New England- United States Of America. The program which relied on ID and clinical pharmacist expertise recorded hospitalized patients receiving two or more parenteral antibiotics, and formulated recommendations for more cost-effective options were shown to have annual savings of 107,637$ [14]. In this research project, we are analyzing the effect of AMS implementation, one year before its initiation, one, and two years after the initiation of AMS in two different hospitals [A, B] in Amman- Jordan. The two chosen hospitals had different approaches to AMS implementation. Hospital “A” restricted some antibiotics to be prescribed only by an ID consultant, or an oncologist, or a pulmonologist, in addition to listing internal guidelines for treating common infectious diseases. Hospital “B”, on the other hand focuses only on restricting a list of antibiotics and auto-stop all antibiotics after seven days of prescribing them. The results of the study showed that these different approaches led to differences in the outcomes of the program.

A systematic effort toward building a program that optimizes antimicrobial use requires serious intentions and hard work. There are mainly three reasons to support those efforts which can be used as well as strategic goals of the antimicrobial stewardship program. They are mainly decreasing the development of resistance, enhance patient safety, and decrease cost [15]. Organizations should use those strategic goals to design parameters to measure the outcomes of the AMS. Defined Daily Dose [DDD] is one measure of the cost of antibiotics use and reflects the consumption of antibiotics. It is promoted by World Health Organization [WHO] and is used widely in hospital settings [16]. The other measure is the change in antibiotic susceptibility and can be measured using an antibiogram; which is a periodic summary of antibiotic susceptibility in an organization or a hospital released by the microbiology lab department or infection control department [17]. While AMS outcomes in Hospital “A” were measured using DDD/1000 patients and antibiogram charts, Hospital “B” relied only on antibiogram charts.

Percentages of ESBL *E. coli* and *Klebsiella* numerical decrease in both hospitals, is a positive sign that they might be going the right way when it comes to implementing AMS. Although, the average percentage of ESBL infection in both hospitals was 43.7%, which is elevated when being compared to other countries. For instance, a German study showed that the proportion of ESBL ranges between 10 and 15% [18], while reports from the United States shows a proportion of 4–10% [19], on the other hand, our results were aligned with the results of other studies from different countries like East Africa, Pakistan, and China which reported 42%, 40%, and 46%, respectively [20–22].

MRSA percentage was highly elevated in Hospital “B” after the first year of implementing AMS. This highlights the importance of discussing this issue and implementing solutions to decrease the incidence of MRSA. This elevation is not well understood and requires further studies, although, we recommend measuring the consumption of Fluoroquinolones since some studies showed a clear correlation between overuse of Fluoroquinolones and the incidence of MRSA [23, 24]. In case an increase in FQ consumption is appeared, restricting those antibiotics is recommended and can decrease the incidence of MRSA. The percentage of MRSA infection in Hospital “A” (24.9%) is comparable to the percentage of MRSA infection in the University of Jordan’s Hospital in the orthopedic surgery department which was (30.4%) according to a study conducted by Zuhdi O. Elifranji et al. [25].

VRE percentages in Hospital “A” decreased over the three years of this study, which is also a good feedback indicating that there is a possibility that this hospital is doing well in implementing AMS. Many studies have shown a correlation between piperacillin overuse and incidence of VRE infection [26], although the change in piperacillin consumption according to DDD results was not significantly changed over the three years of this study. Previous research projects indicate that the prevalence of VRE infection among community acquired UTI is 82.6% which is considered to be a very high percentage compared to the percentage of VRE infection in this study which is 8.9% [27].

While many antibiotics have been shown to have improved susceptibility in Hospital “A”, DDD showed no significant change in the consumption of Fluoroquinolones, Carbapenems, and Piperacillin- Tazobactam, Cephalosporins, and Colistin, while a significant change in consumption was observed among Anti-MRSA antibiotics.

Antibiotic susceptibility has proven to be enhanced during through implementation of the AMS program. In Hospital “A”, after one year of the implementation, six cases have increased susceptibility significantly, and two cases decreased in susceptibility significantly. The second year had shown three cases to be increased significantly in susceptibility and only one case decreased in susceptibility. Even better results were shown in Hospital “B” since after one year of the implementation, seven cases improved significantly and 16 decreased significantly in susceptibility, while in the second year a dramatic improvement occurred since 20 cases improved in susceptibility significantly and only seven cases decreased in susceptibility significantly. This indicates a great improvement in the susceptibility profile of antibiotics in both hospitals when implementing AMS program.

Components of AMS, include educational efforts, pharmacodynamics dose adjustments, use of a computer-assisted medical decision-making system, adaptation to locally customized guidelines, and conversion from parenteral to oral therapy [28]. Education, which is the cornerstone of improving the antibiotics prescription process plays no role in Hospital “A” or Hospital “B”. In addition to that, no computer-assisted medical decision-making system was used in both hospitals, and no conversion from parenteral to oral policy. On the other hand, Hospital “A” has customized local guidelines for most common infectious diseases, while Hospital “B” did not develop any. Both hospitals continuously adjust antibiotics regimens according to cultures, adjust doses according to kidney function, and optimize doses according to MICs of pathogens. These efforts were mainly held by clinical pharmacists, although there were no measured parameters to follow-up the effect of those efforts on AMS. All that highlights vital recommendations to enhance AMS in those hospitals including; education that should be prioritized and regularly assessed, and to focus more on computer-assisted medical decision-making systems to optimize the process of antibiotics choosing and dosing. Most health authorities recommend that the core multidisciplinary stewardship team should include ID and a clinical pharmacist with ID training [29]. The optimal antibiotic stewardship team would also include a clinical microbiologist, information systems expert, hospital epidemiologist, and infection control, practitioner. CDC has emphasized the importance of appointing a pharmacist leader among the stewardship team whose major responsibility is working to improve antibiotic use [17]. Several studies showed that structuring the antibiotic stewardship team in this way proved to be successful in reducing antimicrobial use, hospital stay, and costs [30–32].

Thus, ID and clinical pharmacists should not only be included in the team but also, should play their roles efficiently. In Hospital “B” for example, we recommend putting extra effort into customizing local guidelines and running policies to convert parenteral antibiotics to oral whenever possible to decrease unnecessary adverse reactions and costs associated with infusion and longer hospital stays. AMS program requires frequent optimization and follow-up. This can be achieved using two main pathways; feedback strategy which relies on report writing of patients, and pre-authorization which is the requirement of approval of an infectious disease physician or infection disease pharmacist to prescribe certain antibiotics for selected patients. Hospitals “A” and “B” apply the pre-authorization method, although no proper documentation of patients receiving parenteral antibiotics inside the hospital setting.

Through this study, multiple recommendations are raised to improve AMS outcomes; firstly, both hospitals should regularly monitor and document the safety profile of antibiotics received in the hospital setting, while Hospital “B” should consider reporting DDD. Secondly, the two hospitals are strongly encouraged to actively improve education efforts in their AMS program, especially since it is highly associated with improved outcomes in the early stages of AMS implementation [33]. Thirdly, clinical pharmacists are vital to the success of the AMS program in any organization; they are the drug experts and can play a major role in educating staff regarding proper prescription and administration of antibiotics, monitoring antimicrobial use, and infection control [34]. Therefore, more focus should be made to empower their role, for instance, only five clinical pharmacists work at Hospital “A” and two at Hospital “B” can be inadequate to improve AMS outcomes, given the size of the hospitals. In addition, parameters should be created to measure clinical pharmacists’ role using daily reports of interventions made. Conversion from parenteral to oral therapy is vital to decrease hospital stay and reduce cost and adverse reactions associated with parenteral administration. Therefore, both hospitals should work on creating policies to guide healthcare providers to convert from parenteral to oral whenever possible. Simple and feasible guidelines can optimize the process of conversion from parenteral to oral therapy more smoothly and clinical pharmacists have a crucial role in moderating this process [35]. Finally, some antibiotics have decreased susceptibility throughout the program, thus, extra caution in prescribing those antibiotics, and considering listing them as restricted antibiotics might lead to preventing more serious resistance like Carbapenem-resistant Enterobacteriaceae and MRSA.

## Conclusion

Many barriers can hold hospitals’ administrations and staff from putting effort into succeeding in the antimicrobial stewardship program. Although, the implementation of this program helped in improving antibiotic susceptibility profile, decrease consumption of a limited group of antibiotics, and decreased the incidence of multidrug resistance. Authors recommend participating hospitals to put an extra effort into implementing this program in their hospitals by recruiting extra ID physicians, ID clinical pharmacists, and ID nurses, besides focusing on enhancing the educational program provided to the hospitals’ staff to increase awareness of antibiotics handling methods, plus enhancing monitoring of this program through assessing outcomes including DDD and the antibiograms.

## Data Availability

The datasets used and/or analyzed during the current study are available from the corresponding author on reasonable request.
